# Habitat-associated Genomic Variation in a Wall Lizard from an Oceanic Island

**DOI:** 10.1093/gbe/evad193

**Published:** 2023-10-20

**Authors:** Richard P Brown, Hui Sun, Yuanting Jin, Carlo Meloro

**Affiliations:** College of Life Sciences, China Jiliang University, Hangzhou, P.R. China; School of Biological and Environmental Sciences, Liverpool John Moores University, Liverpool, United Kingdom; College of Life Sciences, China Jiliang University, Hangzhou, P.R. China; College of Life Sciences, China Jiliang University, Hangzhou, P.R. China; School of Biological and Environmental Sciences, Liverpool John Moores University, Liverpool, United Kingdom

**Keywords:** adaptation, GBS, habitat, island, lizard, selection

## Abstract

The lizard *Teira dugesii* exhibits morphological divergence between beach and inland habitats in the face of gene flow, within the volcanic island of Madeira, Portugal. Here, we analyzed genomic data obtained by genotyping-by-sequencing, which provided 16,378 single nucleotide polymorphisms (SNPs) from 94 individuals sampled from 15 sites across Madeira. Ancient within-island divergence in allopatry appears to have mediated divergence in similar species within other Atlantic islands, but this hypothesis was not supported for *T. dugesii*. Across all samples, a total of 168 SNPs were classified as statistical outliers using pcadapt and OutFLANK. Redundancy analysis (RDA) revealed that 17 of these outliers were associated with beach/inland habitats. The SNPs were located within 16 sequence tags and 15 of these were homologous with sequences in a 31 Mb region on chromosome 3 of a reference wall lizard genome (the remaining tag could not be associated with any chromosome). We further investigated outliers through contingency analyses of allele frequencies at each of four pairs of adjacent beach–inland sites. The majority of the outliers detected by the RDA were confirmed at two pairs of these matched sites. These analyses also suggested some parallel divergence at different localities. Six other outliers were associated with site elevation, four of which were located on chromosome 5 of the reference genome. Our study lends support to a previous hypothesis that divergent selection between gray shingle beaches and inland regions overcomes gene flow and leads to the observed morphological divergence between populations in these adjacent habitats.

**Significance**

Divergence of lizard populations can occur *within* small islands and may originate from differences in natural selection between habitats, but there is little or no evidence for this at the genetic/genomic level. Here, we find allelic differences between pairs of beach and inland sites, largely at different locations on a single chromosome of an island lizard. Some of these differences are replicated at different pairs of beach and inland sites. We also found some evidence of elevation effects. Our findings are consistent with the hypothesis of divergent natural selection between habitats leading to genetic divergence in the face of gene flow.

## Introduction

Studies of diversity across island archipelagos occupy a prominent position within evolutionary biology, which date back to Darwin's work on the origin of species (Darwin 1859) and even earlier. Some of the early detailed attempts to understand how divergence had arisen studied the same taxon on different islands (usually species/subspecies), with each island being treated as a homogenous evolutionary unit (Hamilton and Rubinoff 1963; Gorman et al. 1975; Abbott et al. 1977; Diamond 1977). Subsequent research began to show that the assumption of homogeneity was not always correct, with very substantial geographical variation being detected *within* some relatively small islands. This led to a large number of within-island analyses, largely from the late 1980s until the 2000s, many of which focused on island lizards because of the pronounced patterns they often show (e.g., Malhotra and Thorpe 1991, 2000; Bloor et al. 2008; Súarez et al. 2014; see also Juan et al. 2000). One of the main advantages of these island study systems is that they frequently display clear geographical structuring over short distances.

Insights from these within-island studies of divergence have wider application to larger geographical scales. They also help provide a better understanding of the development of island communities containing different ecomorphs. Adaptive responses to different microhabitats seem to partially explain the evolution of sets of species comprising distinct island ecomorphs, although the contribution of spatial isolation is also recognized (Losos 2009; Mahler et al. 2010; Wang et al. 2013).

Detailed studies of individual species have revealed how this spatial isolation is likely to have arisen. Evidence from mtDNA suggests that ancient geological events, such as volcanic activity, have mediated ancient spatial isolation in some cases (e.g., Emerson et al. 1999; Gübitz et al. 2000; Brown et al. 2006; Stoelting et al. 2014), but divergent natural selection has also been inferred when geographical variation in morphology has been found to be correlated with ecological variation, which can be quite pronounced within volcanic islands (e.g., Thorpe and Brown 1989; Brown et al. 1991; Malhotra and Thorpe 1991). In several examples, there is evidence of a combination of divergent natural selection and ancient spatial isolation (e.g., Rees et al. 2001; Thorpe and Richard 2001; Súarez et al. 2014; O’Connell et al. 2021), although there are a few cases, such as the study species here, where ancient spatial isolation does not appear to have contributed to morphological variation.

We recently identified an example of replicated morphological divergence between two specific habitat types in the lizard *Teira dugesii* within the Portuguese Atlantic island of Madeira, in the absence of historical or current interruptions to gene flow (Brown et al. 2023). The habitat types were gray Atlantic shingle beaches, with no vegetation, versus neighboring inland areas that were well vegetated. *Teira dugesii* therefore appears to provide a suitable model for within-island diversity *without* population isolation. The sample design in the Brown et al. (2023) study was aimed at comparing the two habitat types and analyzed only matched pairs of sites at four localities. Here we were able to add more individuals from seven additional localities across the island for improved analysis of environmental effects.

We first examined whether any of the genetic diversity within *T. dugesii* emanated from ancient vicariance events which could lead to generalized divergence across all loci. Justification for this hypothesis was provided by: 1) patterns previously described on the neighboring Canary Islands (Brown et al. 2016, and references therein) and 2) Madeira's physical characteristics and relatively ancient subaerial appearance (5.6–7.0 Ma: Ramalho et al. 2015) with a likely colonization time <3 Ma after island appearance (Brehm et al. 2003) providing a suitably long timeframe for within-island evolution. The only previous study with island-wide sampling of *T. dugesii* identified subtle elevation-related variation in morphology (Báez and Brown 1997). Brehm et al. (2001) described some allozyme variation across three sites within Madeira, although they did not subsequently detect any clear mtDNA structuring between four samples from similar parts of the island (Brehm et al. 2003). Our second and most important aim was to test the hypothesis of candidate loci under selection that were associated with environmental variation. The addition of more sample sites allowed us to examine beach/inland habitat divergence as well as carry out a preliminary test of elevation effects.

Genotyping-by-sequencing (GBS) data were used to test the hypotheses about divergence within *T. dugesii*. We analyzed general genomic structuring across all single nucleotide polymorphisms (SNPs) and used redundancy analysis (RDA; Rao 1964; Legendre et al. 2011) to test environmental associations in the SNPs found to be candidates for selection. We were also able to use a recently published genome from a quite closely related wall lizard (genus *Podarcis*) to help identify/locate these SNPs. Despite arguments against using reduced representation genomic analyses (such as GBS) to detect selection (Lowry et al. 2017), we showed that they can provide valuable insights into genomic regions under selection, supporting their application (Catchen et al. 2017).

## Results

### GBS Data

**Fig. 1.**
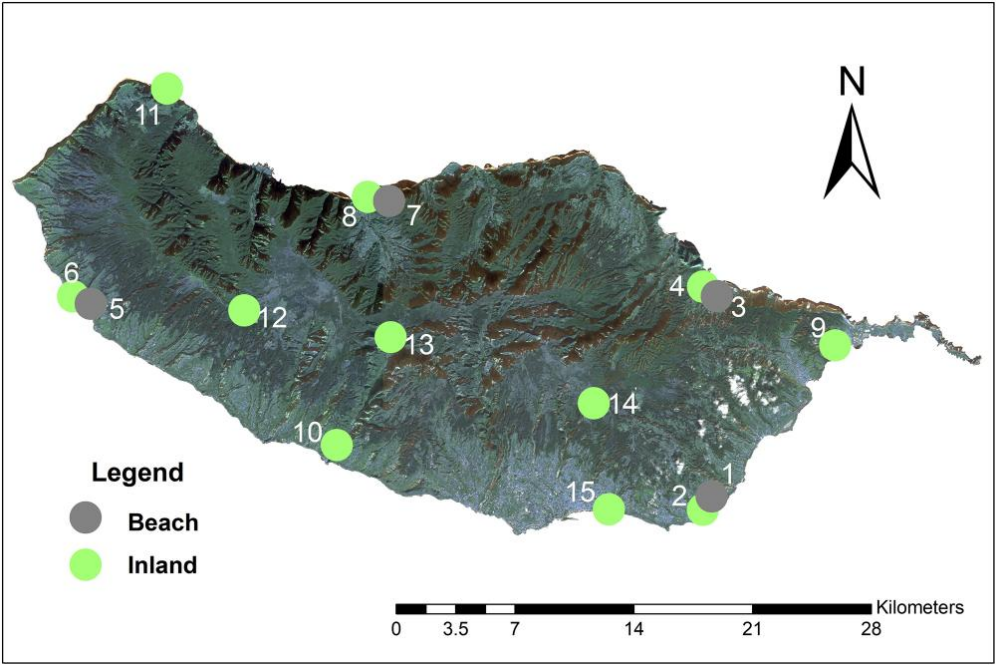
Map of Madeira showing the sample sites numbered 1–15. Circles with different colors indicate locations of inland sites and beach sites. Matched pairs of sites are: 1/2, 3/4, 5/6, and 7/8.

We retained 94 individuals for analysis, covering all 15 sample sites (fig. 1; supplementary table S1, Supplementary Material online). After filtering, the all-SNP data set contained 16,378 SNPs. The thinned-SNP data set comprised 4,466 SNPs (i.e., equal to the number of sequenced tags containing SNPs). The mean number of SNPs missing per individual was 16.2% (standard deviation 0.074; see supplementary table S2, Supplementary Material online).

### General Population Structuring

**Fig. 2.**
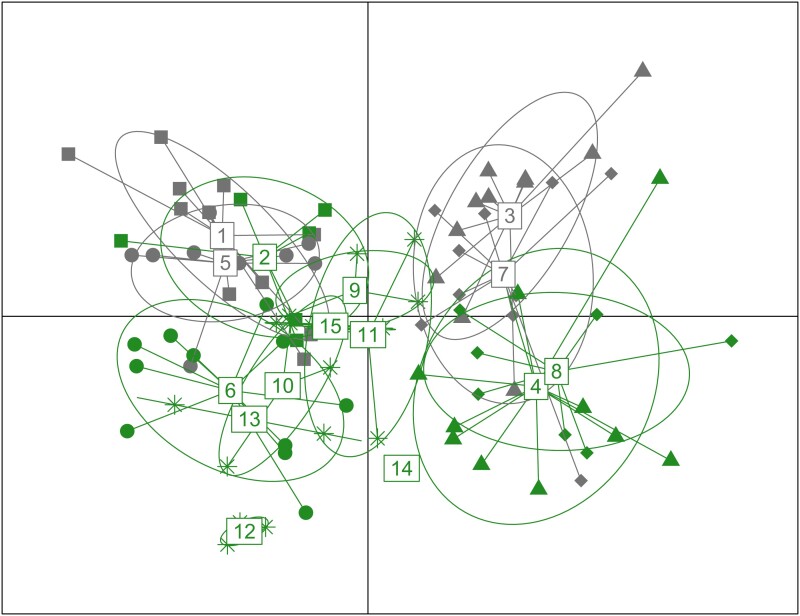
Plot of the first two discriminant function scores from across the 15 sample sites, numbered 1–15. Individuals from the four pairs of adjacent sites, 1–8, have symbols specific to their locality and colors that indicate whether they corresponded to beach or inland. Individuals from inland sites elsewhere on the island (9–15) are represented by asterisks. The first discriminant function (abscissa) represents 63.3% of the variation in the PC scores that were input, while the ordinate represents 21.7%.

The find.clusters approach within the Discriminant Analysis of Principal Components (DAPC) did not reveal distinct genomic clusters (all-SNP data set): the number of clusters (*K*) that best-described structuring, using all 93 principal components (PCs), was *K* = 1 (supplementary fig. 1, Supplementary Material online). Nonetheless, we still applied the DAPC with capture site as the grouping factor (rather than the number of clusters), based on five PCs (determined by cross-validation), to explore structuring further. The first discriminant function (DF1) explained 63.3% of the among-group variation while DF2 explained 21.7%. There was evidence of weak spatial clustering with the north coastal sites 3, 4, 7, and 8 being discriminated from the remaining sites on DF1, although these two groups of sites showed some overlap (fig. 2). There was also some segregation on DF2, with beach individuals tending to show more positive values and inland sites generally negative values.

The LEA analysis of population genomic structuring provided similar results (thinned-SNP data set; supplementary fig. 2, Supplementary Material online). The lowest cross-entropy was detected for 1 ancestral population, with highest cross-entropy for 15 ancestral populations. Even when forced to infer admixture assuming two ancestral populations, the analysis did not reveal any geographically coherent patterns which supported the finding of no general geographical structuring (analysis not shown).

Tests of isolation-by-distance using the Mantel test (thinned-SNP data set) were not significant for either the Weir and Cochran *F*_ST_ distances (*r* = −0.1675, *P* = 0.926) or for the chord distances (*r* = −0.1173, *P* = 0.831).

### Tests of Selection

For the all-sites pcadapt analysis of the all-SNP data set, the principal component analysis (PCA) screeplot indicated that *k* = 4 axes represented most variation among individuals (supplementary fig. 3*A*, Supplementary Material online), and so was specified in pcadapt to define the number of PCs retained. Exploration of alternative values *of k* had little impact on outlier detection. Despite considerable overlap, PC1 scores (2.6% of total variance) for individuals from the four beach sites tended to be mainly negative, while those for the inland sites tended to be mainly positive (supplementary fig. 3*B*, Supplementary Material online). This approach detected 129 outlying SNPs from 114 sequence tags. OutFLANK on the same data set identified a total of 40 SNPs from 35 sequence tags. Only one outlier was identified by both OutFLANK and pcadapt leading to a pooled group of 168 outlying SNPs for the RDA.

**Fig. 3.**
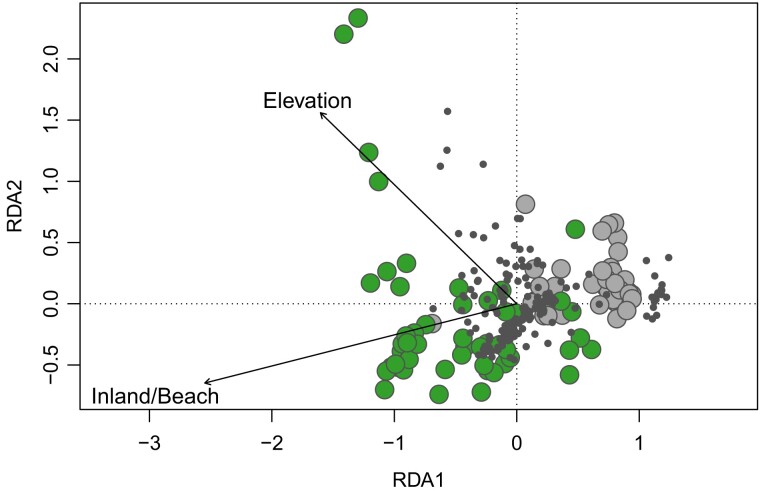
RDA triplot showing the two canonical axes RDA1 and RDA2. The 168 outlier SNPs are represented as filled small dots, beach individuals and inland individuals are given as larger circles with distinct colors. Vectors for the two environmental variables are provided, with “Inland/Beach” showing the direction from beach to inland for the “Habitat” vector. Increasing elevations are indicated by the direction of the “Elevation” vector.

The RDA on the outliers revealed a low adjusted *r*^2^ (0.060): this shows that only a relatively small proportion of outliers were associated with the two environmental variables. The eigenvalue for the first constrained axis was much larger (10.63) than that for the second axis (2.81). Both of these axes were significant (RDA1, *F*_1,91_ = 6.30, *P* < 0.001; RDA2, *F*_1,91_ = 1.66, *P* < 0.001). The permutation test on the marginal effects of each independent variable revealed highly significant effects for habitat (*F*_1,91_ = 7.88, *P* < 0.001) and elevation (*F*_1,91_ = 2.18, *P* < 0.002). These effects are presented as a triplot of individuals, SNPs, and the two environmental variables (fig. 3). It shows evidence of habitat effects on the first constrained axis with elevation effects mainly on the second constrained axis. A total of 23 SNPs from 21 sequence tags were found to be extreme to all other SNPs (defined as them being ≥ 2 standard deviations from the mean loading on the two constrained axes). Seventeen of these SNPs were associated with habitat and six with elevation.

Nucleotide BLAST of the 21 sequence tags (corresponding to the environment-correlated SNPs) against the *Podarcis raffonei* genome provided 20 hits (table 1). Fifteen of the tags (corresponding to 16 SNPs) were found on chromosome 3. The length of this chromosome was 124,660,641 bp and all 15 tags were within the 12.4–43.8 Mb region. Three tags (four SNPs) were found on chromosome 5, one on chromosome 2, and one on chromosome 14. The most interesting result is that 16 of the 17 SNPs that were strongly associated with shingle beach/inland habitat were all located on chromosome 3, with the only exception being a beach/inland correlated tag that could not be reliably assigned to any chromosome. In addition, four of the six sequence tags that were associated with elevation were found on chromosome 5, with the two others being located on chromosomes 2 and 14.

**Table 1:** Information on the 21 Sequence Tags Containing the 23 Outlier SNPs Identified by Redundancy Analysis

Chromosome	Location	Tag Reference	Predicted Gene	Environment Correlate
2	43034808	1,451,115	A-kinase anchor protein 8	Elevation
3	12418672	39,042	Kinesin-like protein kif16b	Beach/inland
3	14097679*	82,322	—	Beach/inland
3	13833659	57,984	Serine palmitoyltransferase 3	Beach/inland
3	16598477	5,676	1-Phosphatidylinositol 4,5-bisphosphate phosphodiesterase	Beach/inland
3	17804868	34,920	Fibroblast growth factor 18	Beach/inland
3	25416490	601	Wd repeat and coiled-coil-containing protein	Beach/inland
3	31116873	81,406	—	Beach/inland
3	36188898	345,306	KH domain-containing, RNA-binding, signal transduction-associated protein	Beach/inland
3	37184764*	1,126	—	Beach/inland
3	37350899	78,478	—	Beach/inland
3	37806784	92	—	Beach/inland
3	38247147	77,921	—	Beach/inland
3	39418336	6,284 (2 SNPs)	Stromal membrane-associated protein 1	Beach/inland
3	41888143	40,410	—	Beach/inland
3	43845201	953	—	Beach/inland
5	15522909	67,027 (2 SNPs)	—	Elevation
5	17326309	189,737	Histone acetyltransferase kat6b	Elevation
5	53029274	17,914	Vesicle transport through interaction with t-snares homolog	Elevation
14	8646921	6,353	—	Elevation
—	—	6,098	—	Beach/inland

Entries in the Chromosome column were those determined from the BLAST search on the *P. raffonei* chromosome, with Location providing the position on that chromosome (an asterisk on the Tag reference indicates where only the R1 or R2 read met the BLAST criteria, not both). Tag references are arbitrary and specific to this study. Predicted genes, where available, were those previously determined for *P. raffonei*. The column “Environment correlate” gives the significant environmental variable revealed by RDA across all sites. Dashes (—) denote where a genomic location and/or a predicted gene was not identified.

**Fig. 4.**
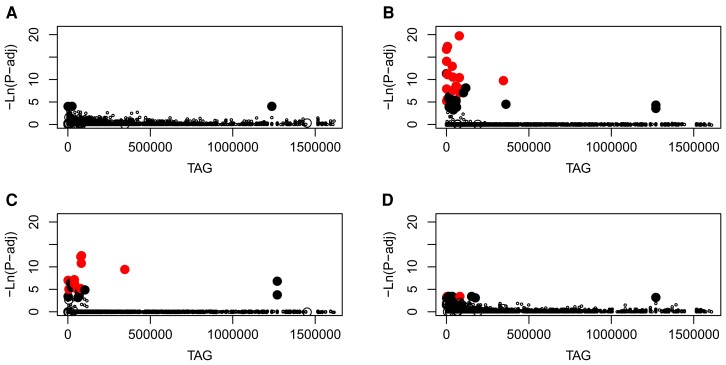
Scatterplots summarizing results of pairwise beach/inland analysis of outliers (matched sites analysis) showing −log*_e_* transformed adjusted probabilities [−ln(*P*-adj)] for SNPs on each sequenced tag (TAG: the TAG axis is categorical and arbitrary, representing TAG reference numbers specific to this study). (*A*) Site 1 versus site 2 (four significant SNPs in the pairwise comparison). (*B*) Site 3 versus site 4 (69 significant SNPs in the pairwise comparison). (*C*) Site 5 versus site 6 (48 significant SNPs in the pairwise comparison). (*D*) Site 7 versus site 8 (17 significant SNPs in the pairwise comparison). Filled red circles represent SNPs that were significant outliers for the specific beach/inland pairwise comparison, as well as for the RDA. Filled black circles are SNPs that were significant for the beach/inland pairwise comparison only. Large open circles are SNPs that were significant for the RDA only. Small open circles represent SNPs that were not significant in either analysis.

SNPs that showed beach/inland divergence in matched pair comparisons of allele frequencies showed overlap with those detected by the RDA (fig. 4) and provided some indications of parallel beach–inland divergence. SNP sample sizes for the four sets of comparisons were: 11,336 for sites 1/2, 12,835 for sites 3/4, 12,171 for sites 5/6, and 11,368 for sites 7/8. Greatest overlap among detected outliers was at the site pairs with slightly larger sample sizes (sites 3/4 and sites 5/6). Sites 3/4 provided the largest sample size (22) and revealed the highest number of significant SNPs (69), which included all but three of the 17 beach/inland SNPs detected by the RDA. The comparisons between sites 5/6 revealed 48 significant SNPs which included 10 of the 17 beach/inland SNPs detected by the RDA. Very significantly, the 2 sets of outliers detected by the 5/6 and 3/4 site comparisons shared 25 common SNPs. The pairwise comparisons based on the smallest sample sizes, sites 1/2 and sites 7/8, provided fewest outliers: 4 and 17, respectively. However, 2 SNPs (on 1 tag) in the 7/8 pair were the same as 2 of the 17 beach–inland outliers detected by the RDA and were also detected by the 3/4 and 5/6 site comparisons.

## Discussion

The current study has identified SNPs that appear to be linked to adaptive variation within an island. We identified a small number of outliers (i.e., showing patterns of divergence expected under selection) and subsequently found that 23 (i.e., 14%) of these were correlated with island environmental variation. Most SNPs (17) were associated with beach/inland habitat variation and located on chromosome 3 of the *Podarcis* reference genome. Hence, we found support for our previous suggestion that divergent selection could explain phenotypic divergence of beach populations in the face of gene flow (Brown et al. 2023) and also identified chromosome 3 as a potential target region for this selection. These findings received some support from replicated patterns of divergent allele frequencies at 25 SNPs between neighboring beach and inland sites (1 km apart) in 2 different parts of the island (16 of which were also detected by the cross-island analysis). Most notable beach–inland divergence was shown by two SNPs on a sequencing tag associated with a stromal membrane-associated protein. These SNPs were detected by the whole-island outliers/RDA and by beach–inland pairwise comparisons at three (out of four) localities. In addition, we found that six SNPs were closely associated with elevation across the island, and four of these were located on chromosome 5 in the reference genome. Together, these findings provide a strong basis for future genome-level studies and ecological field experiments of natural selection on this species that could reveal deeper insights into the dynamics of natural selection on this species.

Across all SNPs, there was no strong evidence of generalized and deep within-island divergence across the *T. dugesii* genome within Madeira. However, multivariate analyses revealed some divergence of samples from an area of the north coast, represented by two pairs of adjacent inland and beach sites. The same analyses revealed similar levels of (overlapping) divergence between beach and nonbeach sites, which is likely to be mediated by a relatively small number of SNPs associated with regions under selection. These findings support rejection of major historical divergence in allopatry. Several lizards from other nearby volcanic Atlantic islands (e.g., Gübitz et al. 2005; Brown et al. 2006; Súarez et al. 2014) and some Caribbean islands (Schneider 1996; Malhotra and Thorpe 2000) show evidence of deep historical divergence in allopatry, with potentially causal geological events having been identified in most of these cases. It is possible that there have not been any physical events on Madeira that could have led to, say, population fragmentation since colonization by *T. dugesii*. Current knowledge of Madeira's volcanic history appears to support this (Ramalho et al. 2015).

The application of RDA to individuals from across the island provided new inferences that extended earlier findings (Brown et al. 2023). Support for divergent beach–inland selection was additionally confirmed for many SNPs by pairwise site comparisons of allele frequencies. The Brown et al. (2023) study demonstrated gene flow between the matched beach–inland populations <1 km apart. This is not too surprising given their proximity, but taken together with the current findings of SNP divergence between these site pairs (despite relatively small sample sizes) then highly divergent selection appears to be supported. It was notable that a greater number of SNPs with significant allele frequency differences were detected for the two pairwise comparisons with slightly larger sample sizes and greater geographical separation (i.e., sites 3/4 and 5/6). It is possible that small differences in one or both of these factors greatly increase the power to detect divergent selection and may explain the lack of similar confirmatory findings between sites 1/2.

Studies that report local adaptive divergence without isolation have grown considerably with improved technologies to obtain genome-wide SNPs (Nielsen et al. 2009; Moody et al. 2015; Dennenmoser et al. 2017; Westram et al. 2018; Llanos-Garrido et al. 2021). Recent findings such as local adaptation between urban and nonurban lizard populations show that underlying genomic divergence can appear in a very short time and also in parallel in different populations (Campbell-Staton et al. 2020; Winchell et al. 2023). The pattern in *T. dugesii* is similar, in part, with the same “beach alleles” being associated with different beaches. As the rate of appearance of identical mutations must be low, we suggest that these alleles are more likely to originate from an island-wide standing stock of genetic variation, rather than represent independent parallel mutations.

Some of the environmental differences between gray shingle beach habitats with no vegetation and the well-vegetated inland areas have been described previously (Brown et al. 2023). The occupation of areas next to the shoreline, including intertidal areas, by *T. dugesii* is most notable for a wall lizard. Davenport and Dellinger (1995) first described these lizards from this habitat and showed that marine isopods were an important component of their diet, unlike lizards found at adjacent inland areas. Differences in ecology between these two areas must be substantial. While it is too early to link genes identified on chromosome 3 with specific functions that may be advantageous in these environments, this will be a useful endeavor for future research. The chromosomal region comprising the outlying SNPs that we were able to detect was localized, but large (31.4 Mb). This is much larger than would be expected under, say, a selective sweep around one locus, so we assume a polygenic response to beach habitats. Nonetheless, it represents around one quarter of the chromosome length and would therefore be a good place to focus future efforts.

Given the limitations of a GBS approach and less intensive sampling from higher elevations, the finding of SNP associations with elevation was perhaps surprising. However, it also provides a significant pointer for future studies. Whole-genome analyses of *T. dugesii* could reveal many more alleles/genomic regions that are associated with elevation or other proxies of climatic variation. As ectotherms, lizards should be highly sensitive to climatic variation and so the detection of SNPs that are under selection might be predicted over a wide range of climates. This has also been confirmed by some other recent studies (Llanos-Garrido et al. 2021; Ruiz Miñano et al. 2022).

In summary, this highly abundant lizard is unusual due to it being found around intertidal areas on Atlantic shingle beaches, in addition to most other habitats on the island of Madeira. We demonstrate the existence of SNPs on a specific region of one chromosome that 1) show patterns of divergence expected under selection, and 2) show allelic variation that is correlated with beach versus inland sites. This further supports the hypothesis that within-island genetic divergence can occur in the absence of isolation. Our study also highlights the utility of reduced representation genome methods for studies of nonmodel organisms. A major argument against use of these methods in studies of selection is that they will have low power due to the small proportion of the genome that is sequenced relative to the lengths of haplotype blocks (i.e., extent of linkage disequilibrium; see Lowry et al. 2017). Here, we have shown that GBS may allow significant initial insights into divergent selection between habitats, supporting its use (Catchen et al. 2017).

## Materials and Methods

### Study Species

*Teira dugesii* is endemic to the Portuguese islands that are administered as the Madeiran archipelago and include Madeira, the Desertas, Porto Santo, and the Selvagem islands approximately 280 km to the south (Brehm et al. 2003). Madeira is the largest and most ecologically heterogenous island within this group, reaching a maximum elevation of approximately 1,862 m.a.s.l. in a surface area of around 742 km^2^. *Teira dugesii* is found across the island and is extremely abundant in most areas.

### Samples and Next-Generation-Sequencing

Tail tips (119) were collected from *T. dugesii* captured at 15 sites across Madeira in July 2019 (fig. 1; supplementary table S1, Supplementary Material online), with field permits provided by the regional government of Madeira (IFCN—DSGFB: capture permit 10/IFCN/2018—FAU MAD issued on April 12, 2018) and ethical approval from the Liverpool John Moores University Animal Ethics committee on May 6, 2019. Sites 1, 3, 5, and 7 were beach sites that were matched with the respective inland sites 2, 4, 6, and 8. All lizards were released unharmed at their site of capture. Tail tips were stored in DNA/RNA Shield (Zymo Research).

Total genomic DNA was extracted from the lizard tail tips and GBS carried out by the Hangzhou Lianchuan Biotechnology Company Ltd (paired-end Illumina reads). Extracted DNA was incubated with the restriction enzymes ApeKI and PstI at 37 °C and the digested DNA recovered using magnet beads. The GBS library was prepared using the NGS Fast DNA Library Prep Set (Illumina, San Diego, CA, USA). The library was purified and electrophoresed on a 2.5% agarose gel and DNA fragments of 350–450 bp were excised and diluted before paired-end sequencing on a NovaSeq 6000 platform (Illumina). Quality filtration was carried out: adapters were removed using AdaptorRemoval v2 (Schubert et al. 2016), and low-quality reads were eliminated using FastQC v0.10.170.

SNPs were called using the GBS-SNP-CROP v. 4.1 pipeline (Melo et al. 2016) which is suitable when there is no reference genome. We selected an individual from site 6 (6.08: supplementary table S2, Supplementary Material online) with a large number of reads to create a mock reference. An initial run of the pipeline allowed assessment of the number of missing SNPs per individual. Any individuals with >31% SNPs missing were eliminated before the definitive final run (these individuals appeared to be randomly distributed across sample areas, which suggested this did not introduce bias). At step 4 of the pipeline (i.e., assembly of the mock reference), the options that were changed from their defaults were: a PEAR *P*-value of 0.01, a PEAR parameter of 100, and minimum acceptable length of a mock reference cluster of 100. At step 5 (alignment of reads to mock reference), all default settings were used except for optional setting for SAMtools view (-opt “-m1 -s156”). Defaults were used at alignment step 6 (identification of variants) and step 7 (filtering of variants) except that a maximum average depth of an acceptable variant was specified as 50. Resultant SNPs and their positions/contigs were output as a VCF file. SNPs were examined using VCFtools and any that showed major heterozygote excess were removed (under the criterion of sufficient heterozygote excess to cause deviation from the Hardy–Weinberg equilibrium at the 1% significance level). The data set containing all SNPs is referred to as “all-SNP.” A thinned data set (“thinned-SNP”) was also created by sampling one SNP per sequence tag from the main data set.

### Geographical Structuring

We used adegenet v.2.1.8 (Jombart 2008), a package written for R (R Core Team 2022), to examine the number of clusters and divergence within the complete data set through application of the DAPC function and related procedures. The aim was to examine generalized genomic spatial structuring that could have arisen from historical or current restrictions to gene flow. The complete data set was used because the analyses within DAPC are suited to dimension reduction of nonindependent variables. The number of clusters within the data was determined using the find.cluster function, using all PCs. The xvalDapc cross-validation procedure was subsequently used to specify the number of PCs to be input into the final analysis.

Genetic structuring was also explored using the R package LEA v.3.10.2 (Frichot and François 2015) on the thinned data set. This provides a similar approach to the well-known program Structure (Falush et al. 2007), but with a different algorithm. It has been shown that estimates of ancestry coefficients are similar to those in Structure but can be more accurate under certain conditions (Frichot et al. 2014). We used the smnf procedure for 1–15 clusters (i.e., ranging from one cluster up to equivalence with the number of sampling locations) with the alpha parameter specified as 100 (other configurations were tested but these did not affect the outcome). This procedure allowed assessment of the number of clusters that best described the data.

Finally, we used a Mantel's test (R package ade4 v. 1.7-20; Dray and Dufour 2007) to further examine geographical structuring by specifically testing for isolation-by-distance. Genomic distances between sites were calculated as both the chord distance *D*_CSE_ (Cavalli-Sforza and Edwards 1967) and Weir and Cochran's *F*_ST_ (Weir and Cockerham 1984) between sites (R package hierfstat 0.5-11), while geographical separation was calculated from latitudes and longitudes. Ten thousand random permutations of the rows/columns of the geographic distance matrix were carried out for each of these tests.

### Outlier Detection

We first determined outlying SNPs using all-sites analyses. These were followed by specific comparisons between the matched pairs of beach/inland sites (described below). The all-sites analyses should provide greater power plus the potential to detect elevation effects. Beach–inland pairwise analyses may have lower statistical power but could provide additional insights, potentially revealing parallel changes between different beach/inland locations.

For the all-sites comparison, we first used the R procedure pcadapt v.4.3.3 (Luu et al. 2017) to identify divergent SNPs, which uses PCA on the individual-by-SNP matrix and identifies SNPs that deviate significantly from the *k* PC axes, where *k* is the number of axes that have been selected to adequately reflect genomic variation among individuals. The optimal value for the number of PCs was assessed from the screeplot of eigenvalues (and examination of PC scores). We applied a false discovery rate of 0.05 and determined outliers from their *q*-values (R package qvalue v. 2.30.0; Storey et al. 2022). In addition, we analyzed variation across all sites using another R package that implements a different approach, that is OutFLANK (v.0.2; Whitlock and Lotterhos 2015). It infers the distribution of neutral *F*_ST_s (SNPs with a minimum heterozygosity of 0.15 were used) and subsequently identifies SNPs that are outliers to this distribution. Individuals were grouped by capture site. The mean *F*_ST_ and degrees of freedom of the expected *χ*^2^ distribution of *F*_ST_s were determined using the thinned data set which should provide greater independence of individual SNPs. Deviation from this distribution was used to define SNPs under selection. The *q* threshold in the analysis was 0.05.

The pcadapt and OutFLANK analyses on all sites provided a subset of SNPs that were candidates for selection, which we then tested against environmental variables using RDA (vegan v.2.6-4 package in R: Oksanen et al. 2022). While the RDA could arguably be applied to all SNPs, we used a more focused approach and only applied it to SNPs that had already been shown to be likely candidates for selection, with the aim of reducing the false discovery rate. Two environmental factors were tested, following previous studies on *T. dugesii* from Madeira: 1) shingle beach or inland site (two-state factor), which was previously proposed to be the basis for divergent selection on morphology (Brown et al. 2023), 2) elevation (expressed as site elevation in meters) which shows some correlation with morphology (Báez and Brown 1997).

Subsequent investigation of the potential effects of selection within each of the four pairs of inland/shingle beach sites, that is sites 1/2, sites 3/4, sites 5/6, and sites 7/8, was carried out using broadly the same protocol to that described by de Jong et al. (2021). First, Fisher's exact test was applied to the two-by-two contingency tables that described the reference/alternative allele frequencies at each site within the matched beach/inland pair (the null hypothesis here is that allele frequencies are not contingent on site). This was computed for every SNP, excluding those that were monomorphic across the specific site pair, so sample sizes differed slightly between comparisons (see Results). Second, log*_e_*-transformations of the *P*-values from the tests were negated and their goodness of fit to expected values determined from an exponential distribution (with the rate parameter equal to the mean of −log*_e_* across all SNPs) was examined using a chi-squared goodness of fit test. Next, the exponential probability distribution function was used (with rate specified by the sample mean) to determine the new probability, *P*(*x* > *x_i_*), where *x_i_* = −log*_e_ P* for the *i*th SNP. Finally, the new probabilities were adjusted for multiple testing using the Benjamini–Hochberg procedure (stats 4.2.2: R Core Team 2022).

### Locations of Genomic Regions Under Selection

Attempts to assign any SNPs that were correlated to environmental variables with genomic regions were performed using an NCBI Nucleotide Blast search (https://blast.ncbi.nlm.nih.gov/Blast.cgi): sequence tags containing the outlying SNPs were compared with the *P. raffonei* (Genbank master accession JAPYJY000000000.1) genome using nblast. This species has the same conserved wall lizard karyotype as *T. dugesii*, that is 2*n* = 38, with 36 single-armed macrochromosomes and 2 microchromosomes, and likely shares a most recent common ancestor with *T. dugesii* around the mid-Miocene (Arnold et al. 2007). R1 and R2 reads for each tag were searched separately due to a lack of overlap. Results were filtered for ≥90% sequence identity and ≥90% query coverage.

## Supplementary Material

evad193_Supplementary_Data

## Data Availability

A vcf file containing the SNPs in this article is publicly available at the Knowledge Network for Biocomplexity (https://knb.ecoinformatics.org/) with doi:10.5063/F1ZC81BV and the sequencing reads are available as NCBI bioproject PRJNA1026057.
