# Supplementary material for: Habitat-associated Genomic Variation in a Wall Lizard from an Oceanic Island
Source: Genome Biol Evol. 2023 Oct 20;15(11):evad193. doi: 10.1093/gbe/evad193 (PMC10637050; doi:10.1093/gbe/evad193)

**Supplementary Material**

**Manuscript: Habitat-associated genomic variation in a wall lizard from an oceanic island**

**Table 1.** Sample site data.

| **Site number** | **Sample size** | **Longitude** | **Latitude** | **Habitat** | **Elevation (m)** |
| --- | --- | --- | --- | --- | --- |
| 1 | 11 | -16.82424W | 32.64595N | Beach | 1 |
| 2 | 6 | -16.82784W | 32.64767N | Inland | 30 |
| 3 | 11 | -16.82059W | 32.76747N | Beach | 1 |
| 4 | 11 | -16.83013W | 32.76629N | Inland | 90 |
| 5 | 9 | -17.22281W | 32.75181N | Beach | 1 |
| 6 | 11 | -17.22619W | 32.75472N | Inland | 25 |
| 7 | 10 | -17.04006W | 32.81117N | Beach | 1 |
| 8 | 7 | -17.04199W | 32.81065N | Inland | 10 |
| 9 | 3 | -16.74641W | 32.73613N | Inland | 40 |
| 10 | 3 | -17.05852W | 32.67843N | Inland | 50 |
| 11 | 3 | -17.16972W | 32.86609N | Inland | 40 |
| 12 | 3 | -17.11827W | 32.74914N | Inland | 1350 |
| 13 | 2 | -17.02594W | 32.73627N | Inland | 590 |
| 14 | 1 | -16.89748W | 32.70320N | Inland | 1270 |
| 15 | 3 | -16.88684W | 32.64693N | Inland | 60 |

**Table 2.** Missing SNPs per specimen after the final run of the GBS SNP-calling pipeline. Individual codes correspond to sample site and specimen number (i.e., “1.02” indicates specimen 2 from site 1). *specimen used for mock reference.

| **Individual** | **Number of missing SNPs** | **Percentage of SNPs missing** |
| --- | --- | --- |
| 1.01 | 2280 | 0.139 |
| 1.02 | 1762 | 0.107 |
| 1.04 | 1939 | 0.118 |
| 1.05 | 4217 | 0.256 |
| 1.06 | 3574 | 0.217 |
| 1.08 | 1029 | 0.063 |
| 1.09 | 735 | 0.045 |
| 1.10 | 3035 | 0.184 |
| 1.13 | 2124 | 0.129 |
| 1.14 | 2426 | 0.147 |
| 1.15 | 2691 | 0.164 |
| 2.04 | 4273 | 0.260 |
| 2.05 | 2126 | 0.129 |
| 2.06 | 4701 | 0.286 |
| 2.13 | 2454 | 0.149 |
| 2.14 | 2050 | 0.125 |
| 2.15 | 2851 | 0.173 |
| 3.01 | 2683 | 0.163 |
| 3.02 | 1272 | 0.077 |
| 3.03 | 1593 | 0.097 |
| 3.05 | 2108 | 0.128 |
| 3.06 | 2823 | 0.172 |
| 3.07 | 3002 | 0.182 |
|  |  |  |
| Table 2 (cont.) |  |  |
| 3.15 | 1899 | 0.115 |
| 3.16 | 4634 | 0.282 |
| 3.20 | 3053 | 0.186 |
| 3.22 | 2477 | 0.151 |
| 3.23 | 1305 | 0.079 |
| 4.01 | 1163 | 0.071 |
| 4.02 | 933 | 0.057 |
| 4.03 | 1199 | 0.073 |
| 4.04 | 2615 | 0.159 |
| 4.05 | 1516 | 0.092 |
| 4.06 | 1408 | 0.086 |
| 4.07 | 1141 | 0.069 |
| 4.08 | 1796 | 0.109 |
| 4.09 | 3417 | 0.208 |
| 4.11 | 3005 | 0.183 |
| 4.12 | 1562 | 0.095 |
| 5.01 | 4972 | 0.302 |
| 5.02 | 3003 | 0.182 |
| 5.04 | 4948 | 0.301 |
| 5.07 | 3311 | 0.201 |
| 5.09 | 3278 | 0.199 |
| 5.11 | 4236 | 0.257 |
| 5.13 | 4544 | 0.276 |
| 5.15 | 1667 | 0.101 |
| 5.16 | 2797 | 0.170 |
| 6.04 | 1654 | 0.101 |
| 6.05 | 1651 | 0.100 |
| 6.06 | 1548 | 0.094 |
| 6.07 | 1776 | 0.108 |
| 6.08* | 2053 | 0.125 |
| 6.09 | 5899 | 0.358 |
| 6.10 | 891 | 0.054 |
| 6.11 | 1158 | 0.070 |
| 6.12 | 1432 | 0.087 |
| 6.14 | 1104 | 0.067 |
| 6.15 | 2382 | 0.145 |
| 7.01 | 5452 | 0.331 |
| 7.02 | 2203 | 0.134 |
| 7.03 | 1402 | 0.085 |
| 7.04 | 2470 | 0.150 |
| 7.06 | 2922 | 0.178 |
| 7.07 | 1415 | 0.086 |
| 7.09 | 1884 | 0.114 |
| 7.11 | 2248 | 0.137 |
| 7.12 | 2078 | 0.126 |
| 7.13 | 3161 | 0.192 |
| 8.03 | 4287 | 0.260 |
| 8.04 | 2505 | 0.152 |
| 8.05 | 3631 | 0.221 |
| 8.06 | 2711 | 0.165 |
| Table 2 (cont.) |  |  |
| 8.07 | 2006 | 0.122 |
| 8.12 | 2733 | 0.166 |
| 8.13 | 1392 | 0.085 |
| 9.01 | 3061 | 0.186 |
| 9.02 | 4732 | 0.288 |
| 9.03 | 5305 | 0.322 |
| 10.02 | 4359 | 0.265 |
| 10.03 | 3144 | 0.191 |
| 10.04 | 3184 | 0.193 |
| 11.01 | 3499 | 0.213 |
| 11.02 | 4973 | 0.302 |
| 11.03 | 5007 | 0.304 |
| 12.01 | 1447 | 0.088 |
| 12.02 | 2060 | 0.125 |
| 12.03 | 1855 | 0.113 |
| 13.01 | 1657 | 0.101 |
| 13.03 | 2858 | 0.174 |
| 14.01 | 2731 | 0.166 |
| 15.01 | 4363 | 0.265 |
| 15.02 | 3102 | 0.188 |
| 15.03 | 3533 | 0.215 |

**Figure 1.** Number of clusters within the data (from DAPC find.clusters function) and their associated Bayesian Information Criterion (BIC) values, supporting a single cluster.


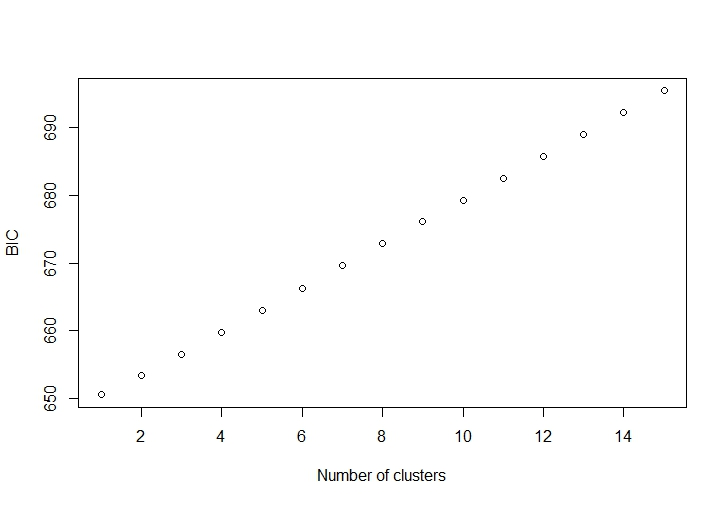


**Figure 2.** Cross-entropy for each value for different numbers of putative ancestral populations, supporting one ancestral population.


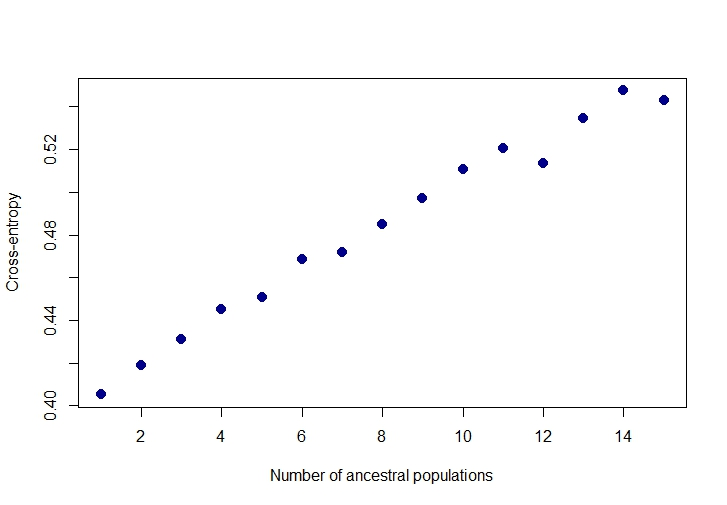


**Figure 3.** Principal component analysis computed by pcadapt. A. Screeplot of the first 15 PCs used to determine the number of principal components used in pcadapt. B. Plot of the first two component scores (PC1: 2.62% of total variation, PC2: 2.12% of total variation) from the PCA computed by the pcadapt package, individuals from beach sites (grey symbols: 1, 3, 5, 7) and inland sites (green symbols: 2, 4, 6, 8) have solid symbols with matched pairs having the same symbol type. Individuals from the other six inland sites (9-15) have site-specific green symbols.

A.


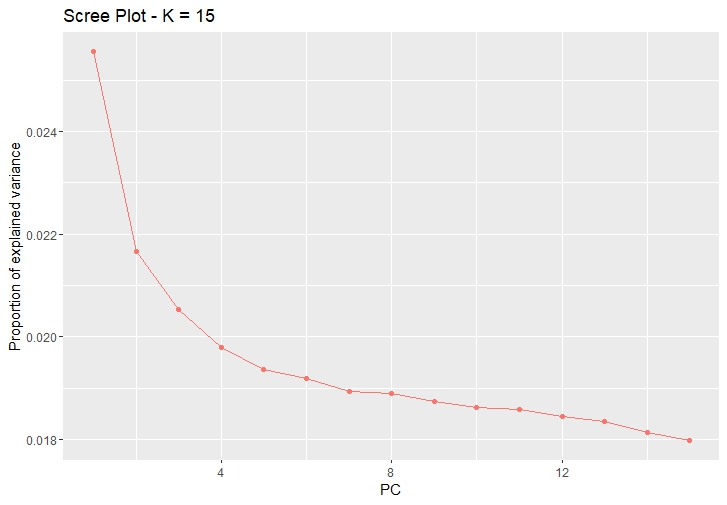


B.


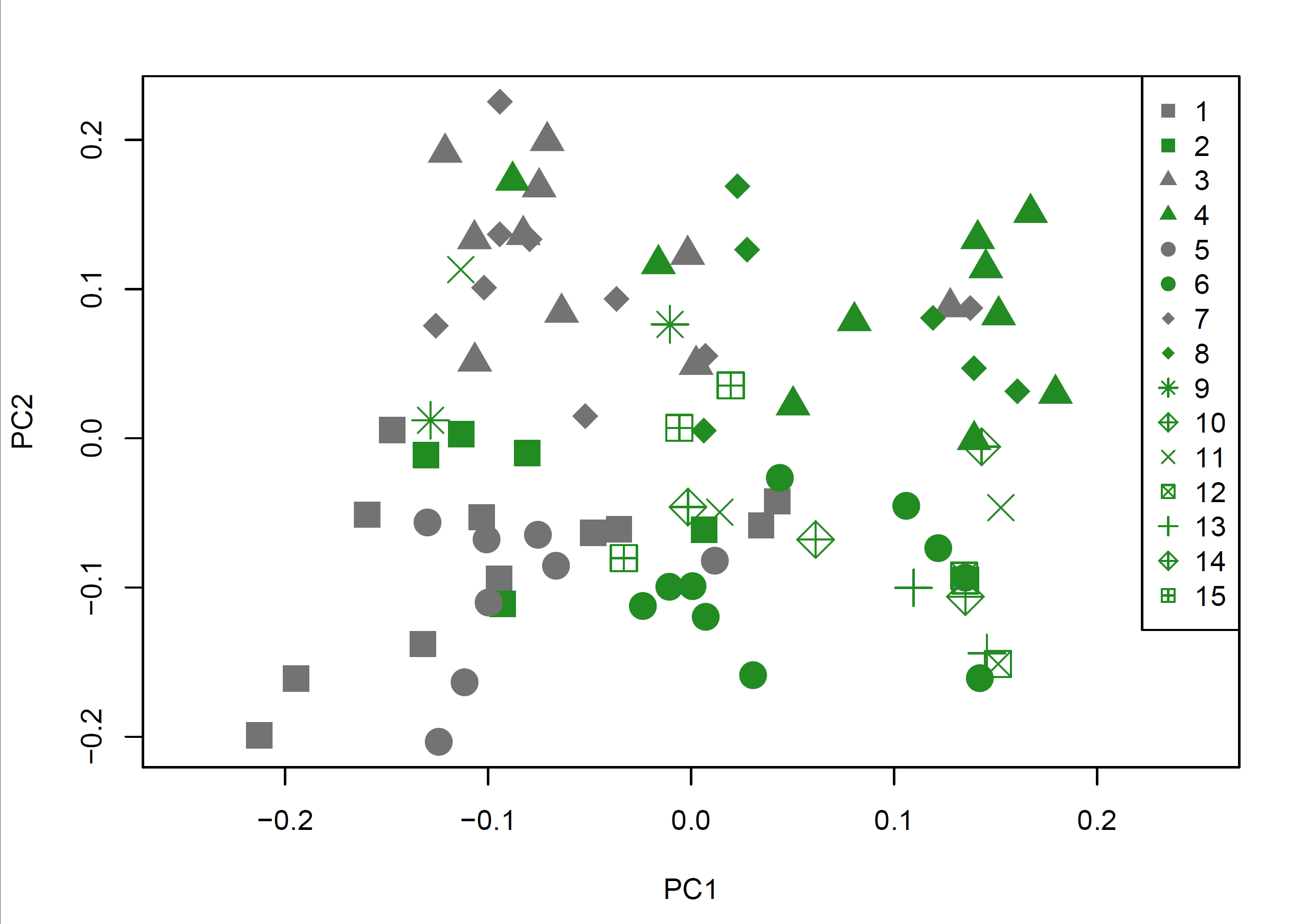

Supplement: evad193_Supplementary_Data [file evad193_supplementary_data.docx]
